# Exploring Cellular Stress Response and Chaperones

**DOI:** 10.3390/cells8050408

**Published:** 2019-05-02

**Authors:** Alessandra Stacchiotti

**Affiliations:** 1 Anatomy and Physiopathology Division, Department of Clinical and Experimental Sciences, Viale Europa 11, 25123 Brescia, Italy; alessandra.stacchiotti@unibs.it; Tel.: +39-030-3717478; Fax: +39-030-3717486; 2 Interdepartmental University Center of Research “Adaptation and Regeneration of Tissues and Organs-(ARTO)”, University of Brescia, 25123 Brescia, Italy

**Keywords:** heat shock proteins, endoplasmic reticulum stress, mitochondria, MAMs, autophagy, proteostasis

## Abstract

Since the pioneering discovery of heat shock proteins in *Drosophila* by Ferruccio Ritossa in 1960s, a long and exciting journey has been undertaken by molecular biologists and researchers worldwide. Not only lower organisms like worms, yeast, amoeba, and flies but also eukaryotes share common cellular response signals to stressful conditions that can arise from the outside but also from the inside. Moreover, extraordinary interplay between nucleus and subcellular organelles, and between different organelles, like mitochondria and the endoplasmic reticulum called mitochondria-associated endoplasmic reticulum membranes (MAMs), are involved in aging and human diseases like obesity, diabetes, inflammation, neurodegeneration, autoimmune diseases, atherosclerosis, and cancer. Actually, we know that to hit abnormal proteostasis and lipid exchanges in the endoplasmic reticulum is crucial to best guide effective therapies or discover new drugs. Indeed, restoration or impairment of endoplasmic reticulum shape and function lead to cellular homeostasis by autophagy or to final death generally by apoptosis or pyroptosis. This Special Issue collects current valuable articles or reviews on cellular stress research and each contribution opens a new window for further studies and hypothesis. I hope that readers interested in this fascinating topic may be stimulated to know more and more.

## 1. Endoplasmic Reticulum as a Peculiar Site of Stress Response

In animal cells, the endoplasmic reticulum (ER) is a peculiar perinuclear network composed of tubular membranes and cisternae that drive nuclear signals to the cytoplasm and Golgi complex, and is essential for assembly and secretion of proteins, lipids, and calcium flux [1]. Palade has, for the first time, morphologically characterized it under a transmission electron microscope in association, or not, with ribosomes called rough (RER) or smooth endoplasmic reticulum (SER) [2]. In the field of stress research, ‘chaperone’ is called a protein resident in ER, mitochondria, or in the cytoplasm, able to assist and hamper abnormal protein folding and trafficking when these processes are disrupted or incomplete. In particular, the ER environment, due to its essential role in the production and assembly of secretory and surface proteins, has peculiar resident chaperones, called glucose regulated protein 78 kDa or immunoglobulin heavy chain binding protein (GRP78/BiP) [3], 94 kDa (GRP94), Protein Disulfide Isomerase (PDI), heat shock protein 47 (HSP47), and lectins like calnexin and calreticulin [4,5]. 

Some calcium-dependent chaperones like GRP78 and GRP94 are peculiar glucose-regulated heat shock proteins. However, the seminal discovery of heat shock proteins in heated *Drosophila* salivary glands was due to Ferruccio Ritossa in the 1960s [6]. 

From then until today, incredible progress has been made in research on stress to characterize multiple chaperone families, their functions in lower organisms and in eukaryotes, and the implications this could have for medicine [7,8,9]. Not only heating but also environmental, toxic, inflammatory, and nutritional stressors are able to induce a peculiar evolutionary conserved mechanism called ‘the stress response’ to restore cellular homeostasis. Under unstressful conditions, GRP78 is the master ER chaperone able to maintain inactive three transmembrane ‘sensors’ called inositol-requiring enzyme 1 (IRE1), PKR-like ER kinase (PERK), and activating transcription factor (ATF6) [10]. On the contrary, when stress occurs, due to oxidative damage, chemical, inflammatory, or metabolic changes, misfolded and dysfunctional proteins fill ER and an ‘ER stress reaction’ begins. In particular, GRP78, dissociated by above transmembrane ER sensors, drives their full activation [11,12]. 

The unfolded protein response (UPR) indicates the transcriptional program that promotes specific gene activation along three ER sensors to restore cellular homeostasis [13]. Interestingly, Dominguez-Martin et al. report here that the social amoeba *Dictyostelium discoideum* is a valid new alternative to yeast to study ER stress and mechanisms to recover its functions [14]. Notably, if an acute and time-limited UPR may be an adaptive response, excessive stress leads to a state called endoplasmic reticulum-associated degradation (ERAD). It consists into a retro-translocation to cytoplasm of ER produced misfolded proteins for their definitive degradation by the ubiquitin-proteasome machinery [15]. 

Indeed, during chronic ER stress a ‘resistant’ response occurs, leading to cell death by apoptosis and abnormal calcium signaling from ER to mitochondria. Intriguingly, the peculiar juxtaposition between ER and mitochondria, called mitochondria-associated ER membranes (MAMs), play a crucial role in the pathogenesis of metabolic diseases [16]. 

Among three ER intermembrane resident sensors, IRE1alpha has been mainly involved in regulated death signaling by specific microRNA modulation and direct binding to specific pro-apoptotic activators [17]. In this Special Issue, Park et al. elucidate the UPR branch involving PERK-induced phosphorylation of eukaryotic initiation factor 2 (eIF2α), crucial to antagonize heat stress-mediated apoptosis in mouse embryo fibroblasts [18]. 

Autophagy (called also macro-autophagy) is an evolutionary ‘clearing’ mechanism that removes excessive protein cargo and disrupted organelles in stress conditions and a critical therapeutic target [19]. Intriguingly, autophagy activated after ER stress is beneficial by restoring cellular functions in a calcium-dependent manner [20,21]. 

## 2. ER Stress into the Pathogenesis of Human Metabolic Diseases

It is widely accepted that abnormal ERAD or UPR processes are involved in the pathogenesis of aging and many human diseases, and their recovery offers enormous therapeutic potentiality [22]. 

In the Special Issue, different reviews analyze the importance of the restoration of ER maintenance in metabolic diseases and in the immune function of cells in innate and cellular-mediated disorders [23,24]. 

**Figure 1:**
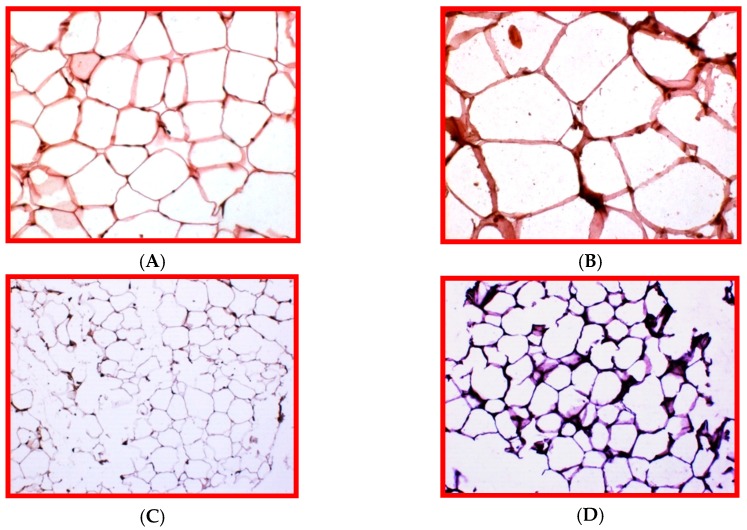
Epididymal white adipose tissue (eWAT) in male C57BL6/J mice fed a standard diet (STD) or a high fat diet (HFD) for 16 weeks. (**A**) Representative histological picture of adipocytes under standard rodent diet; (**B**) Enlarged adipocytes under an obesogenic treatment stained by H&E; (**C**) GRP78 immunostaining is weak in STD fed mice; (**D**) GRP78 immunoreaction is intense in HFD fed mice associated to crown-like structures, a sign of chronic ER stress linked to inflammation. **A**–**B**, Original Magnification= 200×; **C**–**D**, Original Magnification= 100×.

Beside protein unfolding, ER stress is also involved in lipotoxicity and its chaperones are critical in the pathogenesis of non-alcoholic fatty liver disease [25,26]. Obesity analyzed in vitro and in translational rodent models is related to hepatic ER stress and c-Jun-N-terminal kinase (JNK) signaling that affect insulin response [27]. Moreover, in male mice fed a high fat diet, based on 35% lard and 36% carbohydrates for 16 weeks, we documented hypertrophic epididymal white adipose tissue and sustained expression of GRP78 (Figure 1).

Kawasaki et al. similarly outlined ER stress and inflammation in adipocytes, which are then alleviated by oral chemical chaperones [28].

Intriguingly, cancer cells live in a constant stressful microenvironment, due to inflammation, hypoxia, scarce nutrients, and hormones [29]. Therefore, the cancer paradox is that ER stress/UPR whilst preserving the cellular environment, are tumorigenic by reducing apoptosis and inflammation and promoting metastasis [30]. In contrast, ER stress inhibition and crosstalk with the anti-apoptotic factor Survivin is crucial in human colon cancer cells (LS174T) in vitro and in mouse colon under a chronic ER stress [31].

## 3. Conclusions

Promising new evidence indicates that drugs, by acting as molecular chaperones, are effective against protein conformational changes in aging, progeria, diabetes, and other chronic metabolic diseases [32]. However, not only ER but also mitochondria are critical sites of UPR for specific mitochondrial proteins, are necessary to preserve their full function, and to maintain the proper balance between new and aged mitochondria [33].

**Figure 2:**
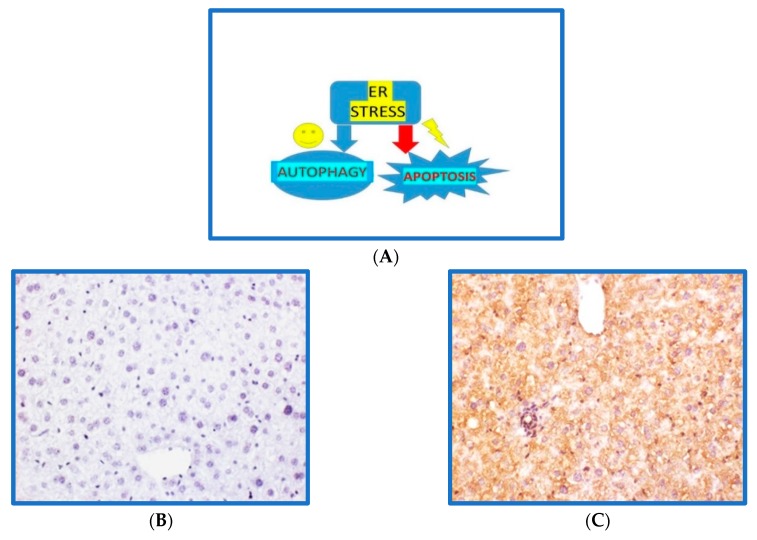
Endoplasmic reticulum (ER) stress response in hepatocytes triggered by a hypercaloric fat rich diet. (**A**) ER stress/ unfolded protein response (UPR) may be a short adaptive mechanism able to restore homeostasis by stimulating autophagy, or it may be chronic and detrimental by triggering apoptosis, or pyroptosis, a peculiar caspase 1-dependent inflammatory cell death. (**B**) Caspase 1 immunostaining negligible in C57BL6/J mice liver fed a standard rodent diet for 16 weeks; (**C**) Caspase 1 immunostaining is intense in C57BL6/J mice liver fed an obesogenic high fat diet for 16 weeks. Original magnification = 200×.

Finally, in Figure 2, we sketched the hepatic interdependence between ER stress and rescue or definitive death, indicated by its influence on autophagy or apoptosis. Moreover, pyroptosis is a typical inflammatory reaction based on caspase 1 expression, and this was evident in the liver of mice fed an obesogenic diet, probably linked to an excessive ER stress response [34].
